# Epigenetic Mechanisms of Resistance to Immune Checkpoint Inhibitors

**DOI:** 10.3390/biom10071061

**Published:** 2020-07-16

**Authors:** Alexandre Perrier, Audrey Didelot, Pierre Laurent-Puig, Hélène Blons, Simon Garinet

**Affiliations:** 1 Centre de Recherche des Cordeliers, INSERM UMR-S1138, Sorbonne Université, Université de Paris, 75006 Paris, France; alexandre.perrier@hotmail.fr (A.P.); audrey.didelot@parisdescartes.fr (A.D.); pierre.laurent-puig@parisdescartes.fr (P.L.-P.); simon.garinet@aphp.fr (S.G.); 2 Department of Biochemistry, Unit of Pharmacogenetics and Molecular Oncology, Georges Pompidou European Hospital, Assistance Publique-Hôpitaux de Paris, 75015 Paris, France

**Keywords:** immunotherapy, immune checkpoint inhibitors, cancer, epigenetics, tumor immune escape, tumor resistance, tumor microenvironment, predictive biomarkers, resistance mechanisms, combination approaches

## Abstract

Immune checkpoint inhibitors (ICIs) have demonstrated to be highly efficient in treating solid tumors; however, many patients have limited benefits in terms of response and survival. This rapidly led to the investigation of combination therapies to enhance response rates. Moreover, predictive biomarkers were assessed to better select patients. Although PD-L1 expression remains the only validated marker in clinics, molecular profiling has brought valuable information, showing that the tumor mutation load and microsatellite instability (MSI) status were associated to higher response rates in nearly all cancer types. Moreover, in lung cancer, *EGFR* and *MET* mutations, oncogene fusions or *STK11* inactivating mutations were associated with low response rates. Cancer progression towards invasive phenotypes that impede immune surveillance relies on complex regulatory networks and cell interactions within the tumor microenvironment. Epigenetic modifications, such as the alteration of histone patterns, chromatin structure, DNA methylation status at specific promoters and changes in microRNA levels, may alter the cell phenotype and reshape the tumor microenvironment, allowing cells to grow and escape from immune surveillance. The objective of this review is to make an update on the identified epigenetic changes that target immune surveillance and, ultimately, ICI responses, such as histone marks, DNA methylation and miR signatures. Translational studies or clinical trials, when available, and potential epigenetic biomarkers will be discussed as perspectives in the context of combination treatment strategies to enhance ICI responses in patients with solid tumors.

## 1. Introduction

Cancer is the second leading cause of death in the world after cardiovascular disease. In 2018, there were 18.1 million estimated new cases of cancer and 9.6 million deaths worldwide [1]. Therefore, cancer remains a major public health problem, with an urgent need for effective and specific treatments. Increased understanding of the immune biology of tumors has led to the development of innovative treatments based on immune stimulation and so-called cancer immunotherapies. Although different strategies have been developed to enhance anticancer immunities, the main drugs used are immune checkpoint inhibitors (ICIs), which specifically inhibit the negative regulators of T-cell activation. The understanding of tumor-induced immune tolerance was the first step of drug development. ICIs on the market target different immune checkpoints as the T lymphocyte receptor CTLA-4 (cytotoxic T lymphocyte antigen 4), the programmed cell death 1 (PD-1) receptor and the programmed cell death 1 ligand 1 (PD-L1). Initially used to treat melanoma, with very encouraging results, ICIs have been tested in many other tumor types with demonstrated clinical efficacy [2]. With this type of treatment, some patients with advanced or metastatic diseases achieve lasting responses that translate into survival benefits never reached with chemotherapy or targeted therapies. For long survivors, ICIs may induce a tumor-specific immunological memory over a long period of time. On the other hand, a significant number of patients have a primary resistance to ICIs, while others with an initial response will develop a secondary resistance and relapse. For example, in metastatic melanoma, high response rates (around 60%) are observed with the combination nivolumab (anti-PD-1) and ipilimumab (anti-CTLA-4), but responses are much weaker with a single agent, 45% with nivolumab and around 20% with ipilimumab, alone [3,4,5,6,7]. In a large trial, progression-free survival (PFS) was 36% with nivolumab-plus-ipilimumab, 29% with nivolumab and 8% with ipilimumab at five years [3]. With pembrolizumab, PFS on the treatments is approximately 35% at one year [8] and around 20% at five years [9]. Many hypotheses have been proposed to explain the nonresponse or escape to ICIs that are either focused on the tumor itself or on the tumor microenvironment (TME): a lack of immunogenic epitopes (low tumor mutational burden), a lack of expression of ICI targets, inflammatory phenotypes, mesenchymal transition, expression of cytokines to reshape the tumor microenvironment that increases—for example, a tumor infiltration by macrophages, high angiogenesis with an expression of the vascular endothelial growth factor (VEGF) and beta-catenin signaling and modulation of the JAK/STAT pathway [10,11,12]. Since immunotherapy is promising to cure cancers of patients with advanced diseases, understanding the causes of resistance is crucial, and finding predictive biomarkers is an objective to select patients and to develop molecules to bypass resistance mechanisms.

Up to now, tumor molecular profiles and tumor mutation loads have brought some information, and expression signatures have been developed, but PD-L1 immunohistochemistry (IHC) remains the sole biomarker in clinics. Due to complex and dynamic interactions between tumors, immune cells and the tumor microenvironment, the finding of a unique biomarker in response to ICIs is unlikely. Here, we focused on epigenetics as a modulator of the response to immunotherapy in cancers and a promising way to overcome resistance.

## 2. The Revolution of Immunotherapy in the Treatment of Cancers

### 2.1. Tumor Microenvironment and Antitumor Immunity

Over the past decade, in vitro and in vivo studies using mice models and human cancers have demonstrated the importance of the immune system to recognize and eliminate transformed malignant cells. Conversely, the immune system also plays an essential role in promoting tumor progression. Understanding the mechanisms of cancer-immune escape is therefore important to design effective immunotherapies.

Antitumor immunity relies on T-cell activation and tumor infiltration by diverse immune cell populations that cooperate to either stimulate or inhibit immune-driven tumor cell death.

The first investigation level is to analyze tumor slides for the presence of immune cells in cancer tissues. Observation has shown high heterogeneity in immune cell density and cell types between tumors, and observations led to the identification of three groups: immune desert, immune excluded and inflamed phenotypes. Differences in terms of the responses to ICIs have been described according to the immune phenotype. In parallel, specific cell subtypes infiltrates are markers of sensibility or resistance to ICIs. For example, it is described in colorectal cancer that the presence of many CD8+ T lymphocytes is a good prognosis factor [13]. In lung cancer, the presence of neutrophils or macrophages has been linked to a low response to immunotherapy [14]. Altogether, immunotherapy is more effective in patients with an inflamed T-cell phenotype [15].

**Figure 1:**
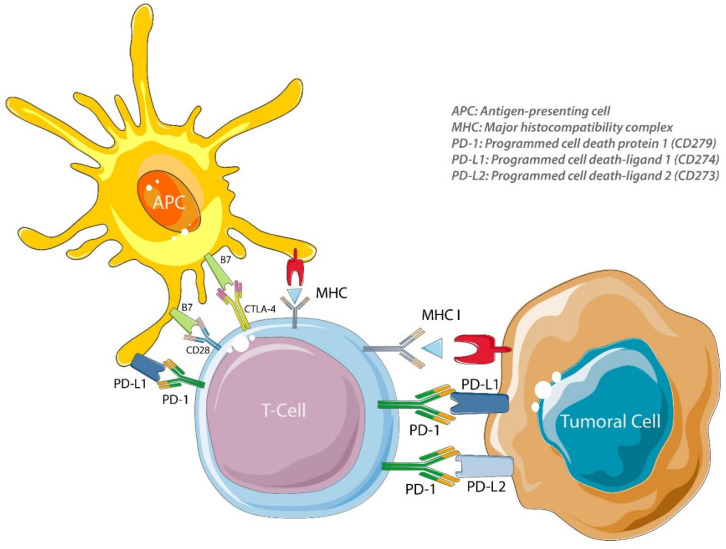
PD-1, PD-L1 and CTLA-4 targets of immune checkpoint inhibitors. APC = antigen-presenting cell, CD28 = cluster of differentiation 28, CTLA-4 = cytotoxic T lymphocyte antigen 4, MHC = major histocompatibility complex, MHC I = major histocompatibility complex class I, PD-1 = programmed cell death protein 1, PD-L1 = programmed cell death 1 ligand 1 and PD-L2 = programmed cell death 1 ligand 2.

At the molecular level, the activation of peripheral immune cells results in a T-cell-inflamed phenotype, with enhanced interferon signaling and a local production of chemokines [16]. As for cell infiltrates, molecular signatures have been developed to select potential ICI responders. The immune response will also depend upon the initiation of tolerance mechanisms, such as the upregulation of PD-L1 and indoleamine 2,3-dioxygenase (IDO) in response to interferon gamma (IFN γ) [17]. Tolerance will turn off T cells and prevent immune tumor control. Indeed, a tumor cell can inhibit the immune system by limiting the action of cytotoxic cells, NK lymphocytes and T lymphocytes. Coinhibitory molecules can be expressed by tumor cells (Figure 1), and tumor cells can promote the infiltration of suppressive cells. Tumor cells are thus able to bypass immune control devices to avoid being attacked and destroyed. “Cold” tumors are characterized by this evasive state. Besides a strict immune escape, tumor cells are often resistant to apoptosis (tumors accumulate mutations during their development, including mutations in proapoptotic genes such as *TP53*, for example), which limits the cytotoxic action of the immune system. By chronic antigen stimulation, T cells can acquire an exhausted phenotype with a diminution of production of IFN γ and interleukin (IL)-2 [18] and the upregulation of immune checkpoint molecules such as PD-1 and CTLA-4 [19] regulated by DNA methylation and alterations of chromatin accessibility [20,21]. As the immune response integrates molecular, cellular and microenvironment modifications, markers should as well integrate data to identify a responsive or nonresponsive phenotype.

### 2.2. Mechanisms of Action of Immune Checkpoint Inhibitors

Key elements of T-cell inhibition mechanisms, called “immune checkpoints” (CTLA-4, PD-1, PD-L1, etc.), can be blocked by “point inhibitors of immune control” or immune checkpoint inhibitors (ICIs). Blocking these brakes reactivate the immune system and, thus, allow the restoration of an effective tumor cells control.

CTLA-4 and CD28 are two homologous receptors expressed by CD4+ and CD8+ T cells [22]. By interacting with two proteins of the B7 family ligands, CD80 and CD86, present on the surface of the antigen-presenting cell (APC) (Figure 1), they regulate T-cell activation oppositely. CTLA-4 interacts with CD80 and CD86 with more affinity and avidity than CD28 and transmits an inhibitory signal to T cells [23,24,25,26,27,28], while CD28 transmits a stimulation signal [29,30]. Furthermore, CTLA-4 is present on the surface of regulatory T cells (Tregs) and contributes to their inhibition function [31,32].

For anti-PD-1 or anti-PD-L1 antibodies, the mechanism of action is based on blocking the interaction of PD-1 with its ligands, PD-L1 and PD-L2 (programmed cell death 1 ligand 2). PD-L1 is more highly expressed than PD-L2 but has a lower affinity for PD-1. Ligands are found at the surface of tumor cells, where their expressions can be induced by type I and II interferons, and at the surface of immune cells, such as macrophages and dendritic cells (Figure 1). In contrast, the PD-1 receptor is mainly expressed by lymphocytes secondarily to their activation. The interaction between PD-1 and PD-L1 or PD-L2 leads to a negative regulation of lymphocytes by inhibiting the signals generated by the T cell receptor (TCR) and the co-stimulation of molecules. Thus, the PD-1/PD-L1 axis is a tumor immune escape mechanism. Using anti-PD-1 or anti-PD-L1 antibodies can thus reactivate tumor-specific lymphocytes within the tumor and allows tumor-specific immune cell death (Figure 1). Beyond the PD-1/PD-L1 axis, many other immune checkpoints and their ligands control, negatively or positively, lymphocyte activation, such as the lymphocyte activation gene 3 protein (LAG-3), which binds to the major histocompatibility complex class II (MHC-II) proteins and to lectins, the T cell immunoglobulin mucin receptor 3 (TIM-3/HAVCR2) and galectin-9, the B and T lymphocyte attenuator (BTLA) and herpesvirus entry mediator (HVEM), the T cell immunoreceptor with Ig and ITIM domains (TIGIT) that binds CD155 and the V-type immunoglobulin domain-containing suppressor of T cell activation (VISTA), for which the ligand is not known [33,34].

### 2.3. History of Immune Checkpoint Inhibitors’ Development and Current Use in Practice

Immune checkpoint inhibitors constitute a breakthrough innovation in the field of oncology. Indeed, the development of ICIs was followed by a complete re-evaluation of therapeutic strategies for unresectable melanoma stages IIIB/IV. One of the first convincing results was obtained in 2010 with the use of ipilimumab, a monoclonal antibody directly directed against CTLA-4 in patients with previously treated metastatic melanoma [35,36]. Given its promising results, with improved overall survival, ipilimumab was approved by the U.S. FDA (United States Food and Drug Administration) in March 2011. In 2014, the anti-programmed cell death 1 (anti-PD-1) antibodies nivolumab and pembrolizumab were approved by the FDA for the same indication. Since then, inhibitors targeting the CTLA-4 and PD-1 immune checkpoints have revolutionized the management not only of melanoma but, also, of non-small cell lung carcinoma (NSCLC) (replacing chemotherapy in the first line for about a third of patients and becoming the standard of care for the second line after chemotherapy failure) [37,38,39,40], renal cell carcinoma (RCC) (in the second line) [41,42], bladder cancers [43] and refractory Hodgkin’s lymphoma [44], with improved survival outcomes in these patient populations (Table 1). There is a major clinical benefit of ICIs among patients with unresectable or metastatic, microsatellite instability-high (MSI-H) or mismatch repair-deficient (dMMR) cancers [45,46]. On the other hand, other localizations like most pancreatic, breast or ovarian tumors seem to be refractory to immunotherapy [47,48]. This led to the development of trials combining immunotherapies, chemotherapy-targeted therapies or vaccines. The opportunity of targeting other immune checkpoints is being investigated, and clinical trials are ongoing to evaluate LAG-3, TIM-3 or VISTA inhibitors. In a near future, a combination of immunotherapies could emerge as treatment options beyond PD-1/PD-L1 and CTLA-4 inhibitors. Selecting the best patients for the best combination remains difficult, and secondary resistances are emerging. Beyond genetics and transcriptomics, we interrogate how epigenetic modification can drive a resistance to ICIs.

## 3. Epigenetic Mechanisms of Resistance to Immune Checkpoint Inhibitors

### 3.1. Epigenetics and Its Roles

Epigenetics studies the nature of mechanisms modifying reversibly, transmissibly (during cell divisions) and adaptively the expression of genes without changing the nucleotide sequence (DNA) [49]. Epigenetic marks, such as DNA methylation and histone post-translational modifications (histone PTMs), participate in the regulation of gene expression and chromatin structures [50,51], allowing or not allowing the transcriptional machinery to access DNA. Several epigenetic mechanisms are involved in resistance to the immune checkpoint inhibitors: the main ones are the modifications of histone marks and chromatin structures, alteration of DNA methylation and changes in miRNA expression levels [52,53,54].

The best-characterized brands are the methyl groups affixed to DNA, as well as various chemical histone PTMs (acetylation, methylation, ubiquitination, phosphorylation, sumoylation, etc.). Histone marks are easily reversible and modify the state of DNA compaction, promoting or limiting the accessibility to genes. Histone methylation at specific lysine residues can be associated with a repressive chromatin state—for example: trimethylations of lysines 9 and 27 of histone H3 (H3K9me3 and H3K27me3)—or associated with an open state of chromatin: such as the trimethylations on lysine 4 and 36 of histone H3 (H3K4me3 and H3K36me3), which are found in promoters’ regions and in the regulatory gene regions, respectively. Lysine acetylation is associated with an open-state chromatin, allowing transcription to occur. Histones PTMs are regulated by enzymes, which can either catalyze mark deposition (“writers”) or their erasure (“erasers”). Histone PTMs can influence chromatin in different ways: signals allowing the recruitment of regulatory proteins (“readers”), modification of the charge of histones (acetylation) and modification of the structure of chromatin. In addition, there are very important cross-regulations. For example, histones marks may inhibit the activity of enzymes, which catalyzes the deposition of other modifications [55]. The modification of histone marks could explain the resistance to immunotherapy treatments and be a promising target.

DNA methylation consists of the addition of a methyl group on cytosine forming 5-methylcytosine at CpG sequences in gene-promotor regions [56]. The DNA methylation marks block transcription and lead to stable long-term repression. This modification of DNA is associated with gene silencing and is carried out by specific enzymes called DNMTs for “DNA methyltransferase”. In humans, there are four: DNMT1, which is a maintenance methyl transferase whose main role is to maintain methylation on the two strands of DNA during replication, DNMT2, whose role is still uncertain and DNMT3A and 3b, which share a strong homology and whose main roles are to add new methylation marks to DNA (“de novo DNA methyltransferase”) [57].

MicroRNAs (miRNAs) are single-stranded, noncoding small ribonucleic acids (RNA) that negatively regulate gene expression at the posttranscriptional level [58]. Their pairing with a target messenger RNA (mRNA) can lead to the inhibition of its translation or to its degradation [59].

### 3.2. Modifications of Histones in Cancers and in Resistance to ICIs

Histone modifications have been described in different studies as factors of cancer progression and resistance to immunotherapy. Histone deacetylases (HDACs), histone methyltransferases (HMTs as EZH2) and the family of BET (bromodomain and extra-terminal domain) seem to be the most involved in the cancer process, but histone acetyltransferases (HATs) and histone demethylases (HDMs) can also be deregulated in cancers [60].

#### 3.2.1. Histone Deacetylases (HDACs)

HDACs are important epigenetic regulators. They remove acetyl groups from N-acetyl lysine amino acid on the tails of histones. They are classified into different classes: I, IIa, IIb, III and IV and impact innate and adaptive immune responses [61,62]. The acetylation of lysines stabilizes decondensed chromatin, which is associated with transcriptional activation. Changes in the expression and/or activity of HDACs have been identified in tumor cells and the disruption of the balance between acetylation (HAT) and deacetylation (HDAC) levels, contributing to altered gene expressions. It was shown that tumors, thanks to epigenetic silencing, could diminish the expressions of cell surface molecules essential to tumor recognition by the immune system. Concerning immunotherapy, the link with PD-L1 expression was clearly established [63]. Therefore, adjustment of the HDAC/HAT balance represents an attractive antitumor strategy, and HDAC inhibitors are tested in clinical trial with ICIs. HDACi may enhance the response to immunotherapy by increasing levels of tumor antigens and the reactivation of proapoptotic genes [64].

#### 3.2.2. Histone Methyltransferases (HMT/EZH2)

EZH2 (enhancer of zeste homolog 2) is an HMT and a member of the polycomb repressive complex 2 (PRC2). It is responsible for histone 3 lysine 27 trimethylation (H3K27me3) [65]. This chromatin mark results in gene silencing and is involved in developmental regulation [66,67]. EZH2 is overexpressed in many cancers, such as breast, bladder, melanoma and prostate cancer [68,69]. In cancer, EZH2 seem to be implicated in cell proliferation and invasion, as well as metastasis. For example, EZH2 is associated with a poor outcome in breast cancer [70,71,72,73,74]. In several cancers, including melanoma, EZH2 is overexpressed or activated by mutation, leading to silencing genes associated with antigen presentation or tumor-suppressor genes [75,76]. A high expression of EZH2 is inversely associated with the tumor infiltration of CD8+ T cells [77]. EZH2 has also been shown to play an important role in the differentiation of Treg cells that suppress immune responses [78]. A recent study found that EZH2, in hepatoma cells, can suppress PD-L1 expression by directly upregulating H3K27me3 levels on the promoters of CD274, which encodes PD-L1 and interferon regulatory factor 1 (IRF1), an essential transcription factor for PD-L1 expression [79]. Many arguments link EZH2 expression to tumor immunogenicity, suggesting that interfering with EZH2 expression could modulate the response to ICIs.

#### 3.2.3. Histone Reader Proteins (BET)

Histone reader proteins bind to structural determinants of histone. By this recognition, the histone code is translated into a functional action. BET family proteins (bromodomain and extra-terminal domain) are histone reader proteins that can bind acetylated histones and modulate the transcription of genes with an immune function. In cancer cells, inhibition of the BET family reduces cytokine production, nuclear factor-kappa B (NF-kB) activity and PD-L1 expression, while increasing natural killer (NK) cell-activating ligands [80,81,82,83,84]. In humans, the BET protein family includes four members: BRD2, BRD3, BRD4 and BRDT. BRD4 is a ubiquitous BET protein involved in many physiological processes. In cancer, BET proteins regulate chromatin remodeling and promote tumor-associated inflammation. Small molecule inhibitors, shown to act as immunomodulatory agents, have been developed and could be combined with immunotherapies to enhance response rates. In ovarian cancer, BRD4 inhibition was shown to reprogram tumor-infiltrating macrophages from the M2-type to M1-type, promoting proinflammatory cytokine secretion and the subsequent activation of CD8+ T cells [85], and, in prostate cancer, BRD4 inhibition was associated with an increased expression of MHC 1 genes by tumor cells, modification of the global gene expression with an activation of antigen-processing networks and an increased CD8+ T cells/Tregs ratio [86]. Altogether, many studies support the potential of BET inhibitors to promote antitumor immune responses. However, it was also shown that BET bromodomain inhibition could have immunosuppressive effects [87] that need to be considered in potential trial testing combinations of immunotherapies with BET inhibitors.

### 3.3. DNA Methylation in Cancers and in Resistance to ICIs

Altered DNA methylation, such as hypermethylation at tumor-suppressor gene promoters and global hypomethylation, was one of the first known processes involved in the genesis of cancer [88,89]. DNA hypermethylation in cancer may also affect the chromatin stability [90]. At methyl CpG loci, CpG-binding reader proteins such as MBD1 (methyl-CpG-binding domain protein 1) and MeCP2 (methyl-CpG-binding protein 2) may recruit HDACs, resulting in the repression of genes involved in immune responses [91]. This is true for PD-L1; the first evidence for PD-L1 downregulation by epigenetic mechanisms was provided by the observation of a relationship between a global hypermethylation measured by methylation arrays and a low PD-L1 expression. In melanomas, the overall hypermethylation of DNA associates with low levels of PD-L1 and correlates with a poor prognosis [92]. Subsequently, studies showed that inhibitors of DNMTs could enhance PD-L1 expression [93]. In human melanoma cell lines, the constitutive expression of PD-L1 is associated with global hypomethylation, especially in intergenic regions and gene introns, but, also, in long terminal repeats (LTRs) and in endogenous retroviruses (ERVs) [81,93]. DNA methylation at ERV regions can block the activation of the IFN signaling pathways and impede immune cells in recognizing tumors. In clear cell renal, ERVs have been shown to encode peptides that elicit T cell and B cell activations [94,95]. Moreover, DNMT1 represses the tumor production of T helper 1 (TH1)-type chemokines CXCL9 and CXCL10 and the impacts on effector T-cell trafficking into the tumor microenvironment [77]. In cancer, DNA methyltransferases such as DNMT1 and histone methyltransferases such as EZH2 are associated with low tumor-infiltrating CD8+ T cells and patient outcomes [77]. The overall hypomethylation of DNA may also contribute to the constitutive upregulation of cytokines such as VEGF and IL-6, which could contribute to the resistance to immunotherapy [93].

### 3.4. miRNAs in Cancers and in Resistance to ICIs

Since they affect the gene expression, miRNAs are involved in many biological processes, including development [96] and tumor formation [97]. MiR-127 was the first epigenetically regulated microRNA reported in cancer [98]. MiRNAs are actors of all the hallmarks of cancer and could be of interest as therapeutic agents [99,100]. Many studies have linked miRNAs and PD-L1 expressions: miR-34a-5p [101], miR-138-5p [102], the miR-200 family [103], miR-424 [104] and miR-513 [105] or PD-1 expressions: miR-138-5p [106,107] and, indirectly, to resistances to immunotherapy (Table 2). By affecting PD-1/PD-L1 interactions, miRNAs modulate T-cell functions [108]. Other miRs such as miR-138-5p were implicated in the regulation of CTLA-4 [107]. Altered miR expressions also act on the tumor immune response through epithelial-mesenchymal transition (EMT) induction. MiRs of the miR-200 family (miR-200a, miR-200b, miR-429, miR-200c and miR-141) repress EMT by inhibiting ZEB1 and ZEB2. Furthermore, they repress PD-L1 expression. Conversely, miR-20b, miR-21 and miR-130b increase PD-L1 expression in colorectal cancer by the inhibition of PTEN, which abolishes PI3K-mediated PD-L1 upregulation [109,110].

## 4. Cellular Mechanisms by Which Epigenetic Alterations Lead to ICI Resistance

### 4.1. Alteration of Tumor Immunogenicity

In the context of cancer, epigenetic alterations participate in the remodeling of the tumor microenvironment and, thus, facilitate its growth and its escape from the immune system. The activation and differentiation of CD8+ T cells are associated with epigenetic changes, loss of the repressive H3K27me3 and acquisition of H3K9Ac and H3K4me3 marks permissive to gene loci-encoding effector molecules such as IFN γ or granzyme B. This permissive signature is generally kept in memory cells. This allows a quick re-expression of the effector molecules during a new challenge with the antigen [137,138]. A similar acquisition of H3K9Ac marks is observed in CD4+ T cells [139]. The constant stimulation of T lymphocytes is associated with a phenomenon of exhaustion of the T lymphocytes [140]. This exhaustion is associated with profound epigenetic changes compared to memory T cells [20]. Combating this phenomenon with epigenetic targets could be a solution [32].

It has been demonstrated that chromatin remodeling is involved in the resistance to ICIs through mutations in the chromatin remodeling complex SWI/SNF (SWItch/Sucrose Non-Fermentable) complexes. PBAF, a chromatin regulatory complex (*PBRM1*, *ARID2* and *BRD7*), regulates chromatin accessibility for the IFN γ pathway within tumor cells, resulting in an increased resistance to T cell–mediated cytotoxicity [141]. *PBRM1* inactivation restores the response to immunotherapy by increasing the tumor immunogenicity [141]. Similar effects are observed with the loss of *ARID1A* [142].

### 4.2. Roles of the EMT in Cancers and a Resistance to ICIs

**Figure 2:**
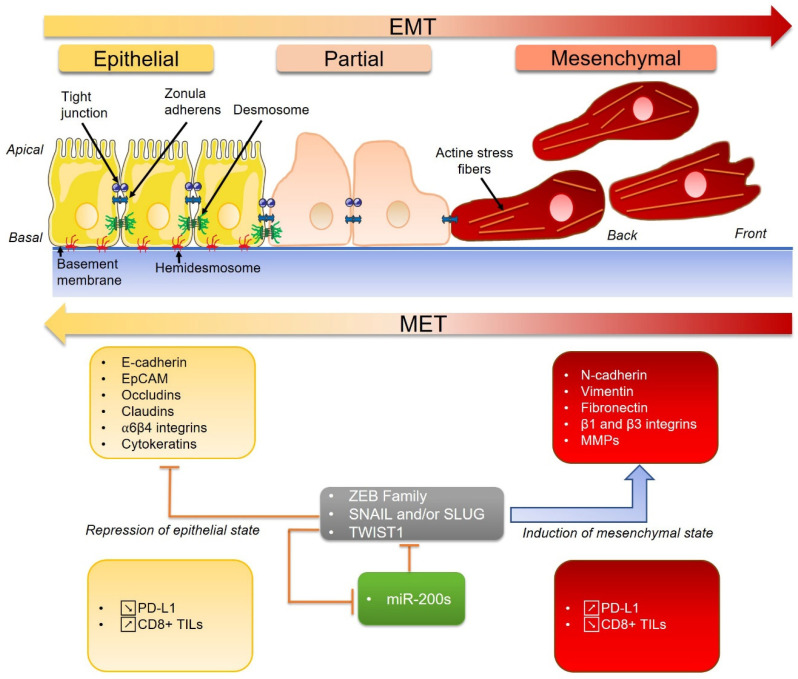
The regulation of the epithelial-mesenchymal transition (EMT) by miR-200s and specific transcriptional factors. EMT = epithelial-mesenchymal transition, MET = mesenchymal-epithelial transition and MMPs = matrix metalloproteinases.

The epithelial-mesenchymal transition (EMT) refers to a dynamic and reversible transition from an epithelial state to a mesenchymal one. Cells undergoing EMT lose their cell-cell adhesion (by a decrease in the expression of cadherins) and acquire new adhesive properties through new interactions with the extracellular matrix by the expression of a specific integrins repertoire (Figure 2). Basal lamina, which borders the epithelium, is degraded thanks to metalloproteinases synthesis [143]. Embryonic transcription factors (TF) such as the ZEB family SNAIL, SLUG1 and TWIST1 are inducers of EMT and may be reactivated in cancer cells (Figure 2). TF upregulations may depend on miR regulations. One major class of EMT-regulating miRs is the miR-200s. They are well-characterized inhibitors of EMT and metastasis that downregulate EMT TFs. Some studies showed that the EMT was linked to PD-L1 upregulation in tumors, demonstrating that the EMT was an important mechanism of immune escape. The EMT and PD-L1 are linked by dysregulation of the miR-200s/ZEB1 axis, a central regulator of the EMT [103]. These findings suggest that a subgroup of patients in whom malignant progression is driven by EMT activators may respond to treatments with PD-L1 antagonists [103].

DNA methyltransferase 3A (DNMT3A) is implicated in EMT-associated metastasis in gastric cancer by repressing E-cadherin through the cooperation of H3K27/H3K9 methylation and DNA methylation [144].

Furthermore, lysine-specific demethylase 1 (LSD1), a histone demethylase implicated in epigenetic regulations of the EMT, in the acquisition of cancer stem cells markers (CSCs) and in therapeutic resistances in breast cancer, could be an interesting target to overcome resistance to ICIs [145]. Based on the identification of an EMT signature, Chae et al. [146] found links between the EMT, exclusion of immune cells, lower infiltration of CD4+ or CD8+ T cells, increase of the expression of multiple immunosuppressive cytokines, including IL-10 and TGF-β, and targetable immune checkpoints (CTLA-4 and TIM-3). The association of the EMT and targetable checkpoints suggests that it could be a marker of sensitivity to the immune checkpoint blockade in NSCLC.

## 5. Epigenetic Biomarkers of Immune Checkpoint Inhibitor Responses

Currently, PD-L1 expression remains the only validated marker in clinics, but this marker lacks specificity and sensibility, and the identification of other predictive markers is needed. Many studies have focused either on genetic alterations or gene expression. In *KRAS*-mutated NSCLC, for example, *STK11/LKB1*-inactivating mutations have been linked to a primary resistance to PD-1 inhibitors [147]. This inactivation of *STK11* leads to a reduced number of tumor-infiltrating lymphocytes (TILs) [148,149,150]. In oncogene-driven NSCLC such as cancers with *EGFR*, *AKL*, *ROS1*, *MET* or other rare fusions, the response to ICIs is globally low, and targeted therapies must be preferred [151,152]. As a source of potential tumor epitopes, the global tumor mutation burden (TMB) was analyzed as a potential biomarker and shown related to an increased response to ICIs [153]. However, technical difficulties and the absence of a consensus cutoff for TMB-high impeded the development of a clinical test. However, the indirect identification of TMB-high tumors through microsatellite instability (MSI) testing or the identification of POLE exonuclease domain mutations is possible to bypass the technical difficulties of TMB testing [154,155]. Other markers involve gene expression signatures such as the type 1 interferon signature [156,157] or the 18-gene tumor inflammation signature (TIS) [158] and tumor microenvironment analyses. Indeed, tumor infiltration by immunosuppressive cells or the exclusion of T cells from the TME may be useful markers to identify responders to ICIs [159]. In melanoma, several studies have reported four groups of patients based on the number of TILs and the level of expression of PD-L1 [160,161,162]. In these studies, the largest group of patients (40% of patients) included those with little or no PD-L1 expression and low TILs, representing most patients failing to respond to PD-1 monotherapy treatment. In locoregional lymph node metastasis, PD-L1+/TIL+ patients had the best outcomes [160].

**Figure 3:**
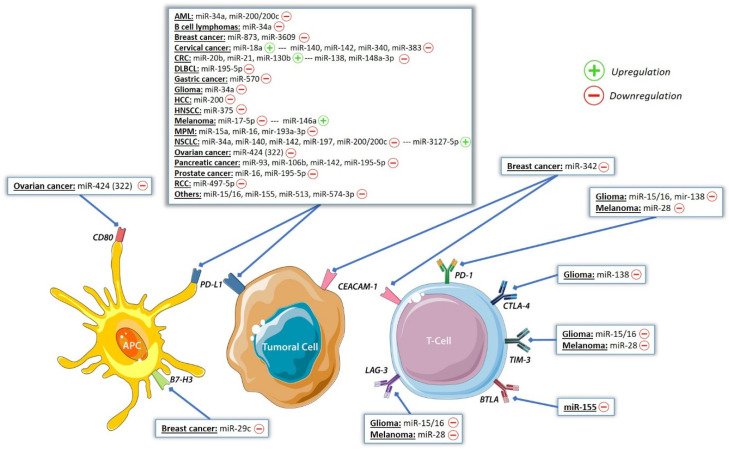
Roles of microRNAs on immune checkpoints. AML = acute myeloid leukemia, CRC = colorectal cancer, DLBCL= diffuse large B-cell lymphoma, HCC = hepatocellular carcinoma, HNSCC = head and neck squamous cell carcinoma, MPM = malignant pleural mesothelioma, NSCLC = non-small cell lung cancer, RCC = renal cell carcinoma, BTLA = B and T lymphocyte attenuator, CEACAM-1 = carcinoembryonic antigen-related cell adhesion molecule 1, LAG-3 = lymphocyte activation gene 3 protein and TIM-3 = T cell immunoglobulin mucin receptor 3.

Given the importance of epigenetic changes in immune response mechanisms, the identification of epigenetic markers is tempting. The most accessible markers are the quantification of DNA methylation marks and miRNAs. MiRs are direct or indirect regulators of PD-L1 expression and of many other immune checkpoints, such as LAG-3, TIM-3, BTLA or CTLA-4 (Figure 3). Concerning, more specifically, PD-L1, EMT-related miRs belonging to the miR-200 and miR-34 families regulate PD-L1 expression by disturbing the ZEB1/miR-200s equilibrium or by direct PD-L1 3′UTR binding [103,130]. The MiR-34s family has also been shown to induce CD8+ TILs in colorectal carcinoma and NSCLC patients [163,164]. Many other miRs have potential predictive values, such as miR-15b, miR-17-5p, miR-34, miR-193a-3p, miR-197 and miR-200c [103,111,113,164,165,166]. It remains to be demonstrated that miRs are not only surrogate markers of PD-L1 expression but drive independent predictive values. A recent study in NSCLC showed that sera miRs profiles could identify responders to ICIs. Authors showed that miR-93, -138-5p, -200, -27a, -424, -34a, -28, -106b, -193a-3p and -181a were increased in the serum of responder patients, which was associated with a significant impact on the outcome [166]. Another group defined an MSC risk (microRNA signature classifier risk) based on the quantification in the serum of 24 miRs. They showed that patients with high-risk MSC did not respond to immunotherapy and suggested that this signature could complement PD-L1 testing for selecting potential responders [167]. Using plasma-derived exosomal miRNAs and miR-seq technology, different miRNA expression profiles were identified between partial-response (PR) and progressive-disease (PD) patients. The authors identified three miRNAs of the hsa-miR-320 family as potential predictors for an anti-PD-1 treatment that all exhibited upregulations in patients with unfavorable responses to the anti-PD-1 treatment [168].

As histone modifications in cancer cells result in the suppression of normal immune responses, histone marks could play a role in predicting responses to immunotherapy. Histone marks as histone 3 lysine 27 trimethylation (H3K27me3) dramatically decrease genes involved in antigen processing and presentation, resulting in low antigenic peptide binding to MHC class 1 molecules and low cytokine production. Histone marks not only in cancer cells but, also, in immune cells might be important to focus on. Indeed, the H3K27me3 is important for T-cell activation and survival [169]. The EZH2 phosphorylation state determines its capacity to maintain CD8+ T memory precursors for antitumor immunity [169]. Detection methods utilize antibodies or mass spectrometry. These tests are not yet ready for routine use; however, proteomic platforms will evolve, and these biomarkers may be used in the future to strengthen patient selections [170].

We discussed earlier that altered DNA methylation profiles in cancer cells or immune cells impinge on tumor-specific immune responses. A few studies pointed out links between DNA methylation and clinics. A DNA methylation signature called the “EPIMMUNE” was identified using DNA methylation microarrays in patients with stage IV NSCLC treated with anti-PD-1. The authors showed that this signature was associated with clinical benefits in patients who were treated with an ICI [171]. Furthermore, the methylation status of FOXP1, a transcription factor involved in the regulation of quiescent CD4+ T cells and the regulation of follicular T helper cells, was found to be predictive of ICI responses. The unmethylated FOXP1 status was associated with improved progression-free and overall survival [171,172]. The authors suggested that FOXP1 could be used in association with validated predictive biomarkers such as PD-L1 staining and TMB to improve patient selection and optimize ICI treatments. However, its predictive value should be evaluated in prospective studies. In 2019, Xue et al. [173] analyzed DNA methylation in 18 cancer types and identified epigenetic signatures based on the methylation status of 269 CpG (corresponding to 191 genes). The signatures were related to the PD-1/PD-L1 inhibitor objective response rates (ORR). In addition, the CpG-based ORR prediction model produced a better performance than the TMB-based model, and, finally, these predictive signatures could allow the identification of potential immune-oncology targets. Another study showed that an increase of histone H3 lysine (27) trimethylation (H3K27me3) and a decrease of E-cadherin associate with a mesenchymal phenotype in nonresponding tumors [174]. In melanoma, low PD-L1 and high global DNA methylation associate with a poor prognosis [92]. Pan-cancer analyses using TCGA (The Cancer Genome Atlas) data demonstrated that genomic global demethylation correlates with immune evasion signatures and affects the clinical benefits of immunotherapy. The authors generated methylome and exome data for 60 samples in an anti-PD-1/PD-L1 cohort in lung cancer and demonstrated that the global low methylation was linked to a high mutation burden and aneuploidy. However, a global low methylation was related to a poor prognosis despite the high mutation load [175]. Since global methylation is relatively stable in cancer cells, its evaluation in clinics as a predictive marker of ICI benefits should be feasible using routine samples. It could allow for the identification of patients that might benefit from a combination of an epigenetic modulation and checkpoint blockade as a potential precision immunotherapy regimen.

To move forward and validate the value of epigenetic marks in clinics, epigenetic analyses should be integrated into trials in association with other known predictors (PD-L1, TMB, MSI, etc.) to establish a global predictive score in the context of personalized medicine and treatment optimization [166].

## 6. Combine Epigenetic Drugs and Immunotherapy to Overcome Resistance

New strategies based on restoring the epigenetic balance to overcome the resistance to cancer immunotherapy are under evaluation. Epigenetic therapies manipulate reversible changes in the tumor and overcome immune and nonimmune mechanisms simultaneously. Epigenetic drugs are FDA-approved, mostly in the context of hematological cancers (Table 3). DNA methyltransferase inhibitors (DNMTi) and histone deacetylase inhibitors (HDACi) are two examples.

**Figure 4:**
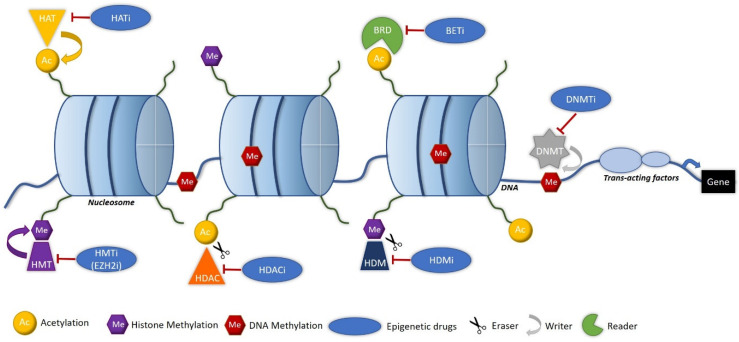
Targets of the epigenetic drugs. BETi = bromodomain and extra-terminal domain proteins inhibitors, BRD = bromodomain, DNMT = DNA methyltransferase, DNMTi = DNA methyltransferase inhibitors, EZH2i = inhibitors of enhancer of zeste homolog 2, HAT = histone acetyltransferase, HATi = histone acetyltransferase inhibitors, HDAC = histone deacetylase, HDACi = histone deacetylase inhibitors, HDM = histone demethylase, HDMi = histone demethylase inhibitors, HMT = histone methyltransferase and HMTi = histone methyltransferase inhibitors.

Currently, the main options being considered are to target histones modulators, inhibit DNA methylation, specifically target miRNAs and the regulatory factors involved in the EMT [60]. Many clinical trials are currently underway to test the epigenetic targets (Table 4). Currently, the drugs target histone deacetylation (HDACi), DNA methylation (DNMTi), histone methylation (EZH2i) and the family of BET readers (BETi) (Figure 4). Combining epigenetic-based treatments with immunotherapy seems to be a promising option to counter the numerous cases of resistance to ICIs. Most of the trials involving combination therapies are in the early phase, and the investigation of their clinical impacts is not yet validated, however. The association of azacitidine and nivolumab produced an encouraging overall survival and response rate in patients with AML [176].

### 6.1. DNA Methyltransferase Inhibitors (DNMTi)

DNMTi are used in myelodysplastic syndrome (MDS) and AML [177]. 5-Azacytidine was shown to inhibit DNA methylation and has been used in the treatment of AML since 1976 [178]. In the context of immunotherapy, there is a new interest in the use of this agent in cancers in which the epigenetic silencing of critical immune regulatory genes has occurred. DNMTi increase tumor antigenicity by several mechanisms, enhancing the expression of MHC molecules and tumor antigens, such as the cancer-testis antigens (CTAs) [179] and ERVs, inducing a state of viral mimicry. Indeed, DNMTI reactivate retroviruses that are normally suppressed by DNA methylation in somatic cells [95]. This allows the recruitment of cytotoxic T lymphocytes into the tumor microenvironment, the modification of cytokine production [180,181] and can also increase interferon signaling. This was first shown in a melanoma animal model [182]. Epigenetic treatments also target the linkage between DNA methylation and PD-L1 expression or T-cell infiltration [93]. In patients with low PD-L1 expression melanoma due to DNA methylation, the silencing of ERVs and IFN pathway gene responses to immunotherapies should be enhanced by combinations with DNMTi such as azacitidine, decitabine [177] or guadecitabine [183,184]. The uses of such combinations would require patient selections [93].

Low-dose decitabine was found to enhance the antitumor effect of the PD-1 blockade in a mouse colorectal cancer model [185]. In a murine ovarian cancer model, the efficacy of anti-CTLA-4 was potentiated with a combination of decitabine [186] and promoted CD8+ TILs. This recruitment of CD8+ TILs was also observed in breast cancer [187]. In human ovarian cancer, Peng et al. [77] found that inhibitors of DNMT1 and EZH2 reactivate the production of T helper 1 (TH1)-type chemokines (CXCL9 and CXCL10), increase effector T-cell tumor infiltrations, slow down the tumor progression and improve the therapeutic efficacy of anti-PD-L1. The epigenetic silencing of TH1-type chemokines is a novel tumor immune-evasion mechanism. Furthermore, DNMTi can reduce T-cell exhaustion by the inhibition of DNMT3a-mediated de novo DNA methylation [188].

### 6.2. Histone Modulators

Currently, there are several histone modulators. The most studied are histone deacetylase inhibitors (HDACi) and histone methyltransferase inhibitors (EZH2i) in cancers. HDACi are studied in different diseases, including noncancerous ones such as inflammatory diseases. Several of them are FDA-approved for cancer: belinostat for peripheral T-cell lymphoma, panobinostat for multiple myeloma, romidepsin for cutaneous T-cell lymphoma and vorinostat for cutaneous T-cell lymphoma (Table 3).

#### 6.2.1. HDAC Inhibitors (HDACi)

Many studies have shown that HDAC inhibitors can reduce tumor growth and promote apoptosis [189,190]. Treatments with HDACi can increase the expression of MHC molecules on tumor cells and the expression of tumor antigens, thus facilitating the action of T lymphocytes [191,192]. HDACi can also increase the production of cytokines and antigen presentations by inhibiting Tregs [193] and increase the NK cell activity by the upregulation of NKG2D (natural killer group 2, member D) [190,194]. Furthermore, histone deacetylase inhibitors demonstrated interesting effects on the reversal of resistance to ICIs in many cancer cells lines [195,196,197] by regulating the expression of several tumor-suppressor genes that are involved in cancer cell apoptosis [198]. Several studies have shown that HDAC inhibitors can restore TP53 protein transcription and allow resistant cancer cells to undergo apoptosis [199]. They also seem to upregulate PD-L1 in melanoma cells and synergize with the PD-1 blockade [63,200]. In lung tumor models, using HDACi romidepsin in vivo increased the response to PD-1 blockade immunotherapy and enhanced T-cell infiltration [201]. In another study, HDACi entinostat enhanced anti-PD-1 therapy by the inhibition of myeloid-derived suppressor cells (MDSCs) in murine models of lung and renal cells [202]. However, HDACi have side effects such as lymphopenia that restrict the immunotherapy efficacy.

#### 6.2.2. Histone Methyltransferase Inhibitors (HMTi/EZH2i)

EZH2 inhibitors cause the blockage of H3K27 methylation. In lymphomas, EZH2 can be activated by different mutations [203], and the use of EZH2 inhibitors allow the selective killing of tumor cells carrying these mutations [204]. As described previously, EZH2i removes the repression of TH1-type chemokines and increases effector T-cell tumor infiltration, reduces the tumor progression and improves the therapeutic efficacy of anti-PD-L1 [77]. Another study found that EZH2 inactivation reversed the resistance to anti-CTLA-4 and IL-2 immunotherapy and suppressed melanoma growth in a consequence of accumulating IFN γ, producing PD-1 ^low^ CD8+ T cells and PD-L1 downregulation on melanoma cells [205].

#### 6.2.3. Histone Reader Protein Inhibitors (BETi)

The inhibition of BET proteins drives an anti-inflammatory effect and reduces dendritic cell maturation [206] and macrophage cytokine production [207]. In ovarian cancer cells, the inhibition of BET decreases PD-L1 expression, tumor-associated dendritic cells and macrophages but increases the activity of antitumor cytotoxic T cells, limiting tumor progression [82]. In a Myc-driven B cell lymphoma, the BETi JQ1 inhibits the PD-1/PD-L1 axis by the loss of CD274 (PD-L1) mRNA production. Moreover, the authors showed that its association with anti-PD-1 is synergistic in a mouse model of MYC-driven lymphoma [208]. In NSCLC, cooperative effects between JQ1 and the PD-1 antibody were found in mice models with a mutation of *KRAS* and deletion of *TP53* [209]. The same molecule was used in a prostate cancer murine model, with similar conclusions [86]. However, BET inhibitors have, as a side effect, reversible neutropenia and thrombocytopenia [210]. Bromodomain proteins are new targets for anticancer treatments. The next challenge will be to identify patients that will benefit from combined immunotherapy and BETi treatments.

Histone acetyltransferase inhibitors (HATi) and histone demethylase inhibitors (HDMi) as LSD1 have also shown antitumor effects on in vitro and in vivo models (especially through the inhibition of the NF-kB pathway) but are less advanced in their development than other epigenetic modulators in association with immunotherapy.

#### 6.2.4. A Promising Target: EMT Regulation Factors

Whether the EMT drives epigenetic modifications or whether epigenetic modifications drive the EMT is a complicated debate. However, both are so closely related that therapeutic interventions that inhibit the EMT will impact epigenetic changes and conversely. LSD1 (protein lysine-specific histone demethylase 1A), also named KDM1A, demethylates a range of important cancer-causing nonhistone proteins, including DNMT1, HSP90 and STAT3, and could be a promising target [211]. Indeed, in breast cancer cells, LSD1 phosphorylation at serine-111 promotes the EMT and changes in the tumor microenvironment to favor an innate, M1 macrophage-like immune response [145]. In vivo, the inhibition of LSD1 combined with chemotherapy reduced the tumor volume and abrogated the mesenchymal signature [145]. In another study, Sheng et al. [212] demonstrated that the depletion of LSD1 in cancer cells increases the repetitive elements expression as ERVs and decreases the RISC components (RNA-induced silencing complex). It led to an activation of type 1 interferon to the stimulation of an antitumor T-cell immunity that restrains tumor growth. LSD1 ablation enhances tumor immunogenicity and T-cell infiltration. In anti-PD-1 therapy refractory mice, the depletion of LSD1 restores the response to the treatment. The authors found an inverse correlation between the LSD1 expression and CD8+ T-cell infiltrations in various human cancers in a TCGA analysis. In light of these developments, LSD1 inhibition could be a promising epigenetic adjuvant therapy to ICIs.

Another option is to target EMT transcription factors such as ZEB or TWIST by siRNA-based (small-interfering RNA) nanoparticle therapies in combination with ICIs. Noman et al. [130] studied the inhibition of ZEB-1 by siRNA silencing and the overexpression of the miR-200 family in human breast cancer cells and observed a strong decrease of PD-L1 expression. More studies should be carried out to confirm the potential of EMT inhibitors as adjuvants of ICIs in subgroups of patients in whom progression is driven by EMT transcriptional factor. More generally, miRNA-based drugs (miRNA mimics or miRNA antagonists) are promising and could be a novel strategy for cancer therapy.

## 7. Discussion and Perspectives

As described in this review, epigenetic changes in cancer and immune cells are implicated in tumor immune escapes and could be important biomarkers to identify responders to immunotherapy, as well as targets to overcome the resistance to ICIs. Some therapeutic targets are clearly identified—namely, DNA hypermethylation and several histone modifiers, such as HDAC; EZH2; BET and EMT drivers (LDS1, TWIST1, ZEB, etc.). Several therapeutic trials are underway, but combinations are still at the early stages of development.

From a broader perspective, the additional toxicity provided by these molecules, in addition to immunotherapy, must not be underestimated. Some epigenetic drugs have been used for a long time with manageable side effects, but adverse effects in association with ICIs have not been extensively studied, especially when treatments are taken over a long period of time. Today, apart from PD-L1, no biomarker has genuinely been really validated. Numerous studies have attempted to validate TMB, molecular signatures and tumor microenvironment analyses as biomarkers to predict ICI efficacy, but none is used in routine care. Epigenetic markers like others need stronger validation. Moreover, immuno-scores taking into account several parameters, including epigenetic marks, would probably be a better way of not only identifying responders to ICIs but, also, patients that would need combination therapies. In addition to their clinical relevance, the routine feasibility and cost issues must be considered. Currently, the investigation of epigenetic marks at a large scale is feasible for research purposes but could be difficult to validate in clinics due to the quantity and quality of tissue obtained in care settings. However, testing platforms are evolving, and technical issues might rapidly be solved. DNA methylation and miRNA expression profiles are good candidates to enhance the clinical values of markers such as TMB, MSI or TILs.

Immunotherapy is not only limited to immune checkpoint inhibitors. One other promising treatment is adoptive cell transfer, which involves modifying immune cells and transferring them back to the patient. It is obvious that epigenetic alterations will also influence the responses to these therapeutic approaches.

Immunotherapy and especially immune checkpoint inhibitors have changed the management of cancer patients for many cancer types. Unfortunately, primary and secondary resistances remain major issues regarding these treatments. Epigenetic alterations have an underestimated role in predicting checkpoint inhibitor efficacies. Moreover, most epigenetic alterations are reversible mediators of primary or secondary resistances to ICIs. This opens the way to the use of combined treatments in subgroups of patients with epigenetic-driven cancers. The good use of immunotherapies is challenging in terms of research, the selection of responder patients, the selection of combinations of drugs, understanding toxicities and co-toxicities, the organization of care, biomarkers and access to testing, the drug circuit and the control of health costs.

## Figures and Tables

**Table 1:** Approved immune checkpoint inhibitors (ICIs) and their indications.

Antibody	Target	Approval Date by FDA	Approved Treatment for Metastatic Cancers
**Atezolizmab**	PD-L1	2016	NSCLC TNBC Urothelial cancer
**Avelumab**	PD-L1	2017	MCC RCC (with axitinib) Urothelial cancer
**Cemiplimab**	PD-1	2018	Cutaneous SCC
**Durvalumb**	PD-L1	2017	Bladder cancer NSCLC
**Ipilimumab**	CTLA-4	2011	Melanoma MSI-H/dMMR CRC Intermediate or poor-risk RCC (with nivolumab)
**Nivolumab**	PD-1	2014	Cervical cancer Classic Hodgkin’s lymphoma Gastric cancer HCC HNSCC MSI-H or dMMR CRC NSCLC Primary mediastinal DLBCL RCC SCC SCLC Unresectable or metastatic melanoma Urothelial cancer
**Pembrolizmab**	PD-1	2014	Cervical cancer Classic Hodgkin’s lymphoma Endometrial carcinoma Esophageal cancer Gastric cancer HCC HNSCC MCC MSI-H or dMMR CRC MSI-H or dMMR non-CRC NSCLC Primary mediastinal DLBCL RCC SCC SCLC Unresectable or metastatic melanoma Urothelial cancer

CRC = colorectal cancer, DLBCL = diffuse large B-cell lymphoma, dMMR = deficient mismatch repair, HCC = hepatocellular carcinoma, HNSCC = head and neck squamous cell carcinoma, MCC = Merkel cell carcinoma, MSI-H = microsatellite instability-high, NSCLC = non-small cell lung cancer, RCC = renal cell carcinoma, SCC = squamous cell carcinoma, SCLC = small cell lung cancer and TNBC = triple-negative breast cancer. All Food and Drug Administration (FDA)-approved indications are for metastatic cancers, except unresectable melanoma for nivolumab and pembrolizumab. CTLA-4 = cytotoxic T lymphocyte antigen 4, PD-1 = programmed cell death protein 1 and PD-L1 = programmed cell death 1 ligand 1.

**Table 2:** MicroRNAs (miRNAs) regulating the PD-L1 expression on cancer cells.

miRNAs	Effects of miRNA on PD-L1 Expression	Cancer Cell Types
**miR-15a**	Downregulating	MPM [111]
**miR-16**	Downregulating	Prostate cancer [112]
	Downregulating	MPM [111]
**miR-17-5p**	Downregulating	Melanoma [113]
**miR-18a**	Upregulating	Cervical cancer [114]
**miR-20b**	Upregulating	CRC [109]
**miR-21**	Upregulating	CRC [109]
**miR-34a**	Downregulating	B cell lymphomas [115]
	Downregulating	Glioma [116]
	Downregulating	AML [101,117]
**miR-93**	Downregulating	Pancreatic cancer [118]
**miR-106b**	Downregulating	Pancreatic cancer [118]
**miR-130b**	Upregulating	CRC [109]
**miR-138**	Downregulating	CRC [119]
**miR-140**	Downregulating	Cervical cancer [114]
	Downregulating	NSCLC [120]
**miR-142**	Downregulating	Cervical cancer [114]
	Downregulating	NSCLC [121]
	Downregulating	Pancreatic cancer [122]
**miR-146a**	Upregulating	Melanoma [123]
**miR-148a-3p**	Downregulating	CRC [124]
**miR-191-5p**	Downregulating	Colon adenocarcinoma [125]
**miR-193a-3p**	Downregulating	MPM [111]
**miR-195-5p**	Downregulating	Pancreatic cancer [126]
	Downregulating	Prostate cancer [112]
	Downregulating	DLBCL [127]
**miR-197**	Downregulating	NSCLC [128]
**miR-200 family**	Downregulating	Lung cancer [103]
	Downregulating	HCC [129]
	Downregulating	Breast cancer [130]
	Downregulating	AML [117]
**miR-340**	Downregulating	Cervical cancer [114]
**miR-375**	Downregulating	HNSCC [131]
**miR-383**	Downregulating	Cervical cancer [114]
**miR-424**	Downregulating	Ovarian cancer [104]
**miR-497-5p**	Downregulating	RCC [132]
**miR-570**	Downregulating	Gastric cancer [133]
**miR-873**	Downregulating	Breast cancer [134]
**miR-3127-5p**	Upregulating	NSCLC [135]
**miR-3609**	Downregulating	Breast cancer [136]

AML = acute myeloid leukemia, CRC = colorectal cancer, DLBCL = diffuse large B-cell lymphoma, HCC = hepatocellular carcinoma, HNSCC = head and neck squamous cell carcinoma, MPM = malignant pleural mesothelioma, NSCLC = non-small cell lung cancer and RCC = renal cell carcinoma.

**Table 3:** Epigenetic drugs approved by the U.S. FDA.

Name of Drug	Synonym	Clinical Name	Class	Approved Treatment	Approval Date by U.S. FDA
**5-Aza-2′-deoxycytidine**	5-Aza-CdR, decitabine	Dacogen ^®^	DNMTi	Myelodysplastic syndrome	2006
**Azacitidine**	5-Azacitidine, 5-Aza-CR	Vidaza ^®^	DNMTi	Myelodysplastic syndrome Acute myeloid leukemia	2004
**Belinostat**	PXD101	Beleodaq ^®^	HDACi	Peripheral T-cell lymphoma	2014
**Panobinostat**	LBH589	Farydak ^®^	HDACi	Multiple myeloma	2015
**Romidepsin**	Depsipeptide, FK-229, FR901228	Istodax ^®^	HDACi	Cutaneous T-cell lymphoma	2009
**Suberoylanilide hydroxamic acid (SAHA)**	Vorinostat	Zolinza ^®^	HDACi	Cutaneous T-cell lymphoma	2006

DNMTi = DNA methyltransferase inhibitor and HDACi = histone deacetylase inhibitor.

**Table 4:** Examples of clinical trials combining immunotherapies and epigenetic regulators.

Drug	Target(s)	Cancer Type	Phase	Status and Enrolment	NCT Number
Azacitidine Pembrolizumab Epacadostat	DNMT PD-1 IDO1	Solid Tumor Advanced Malignancies Metastatic Melanoma	Phase 1/2	Completed (March 2020) 70	NCT02959437
Azacitidine Pembrolizumab	DNMT PD-1	Refractory Acute Myeloid Leukemia (AML)	Phase 2	Recruiting 40	NCT02845297
Azacitidine Entinostat Nivolumab	DNMT HDAC PD-1	Non-Small Lung Cancer	Phase 2	Recruiting 120	NCT01928576
Azacitidine Durvalumab	DNMT PD-L1	Head and Neck Cancer	Phase 1/2	Recruiting 59	NCT03019003
Azacitidine Durvalumab	DNMT PD-L1	Microsatellite Stable Colorectal Carcinoma Platinum Resistant Epithelial Ovarian Cancer Type II Estrogen Receptor Positive and HER2 Negative Breast Cancer	Phase 2	Recruiting 28	NCT02811497
Azacitidine Avelumab	DNMT PD-L1	Recurrent Acute Myeloid Leukemia Refractory Acute Myeloid Leukemia	Phase ½	Recruiting 138	NCT03390296
Azacitidine Durvalumab Romidepsin	DNMT1 PD-L1 HDAC	Lymphoma, T-Cell	Phase ½	Recruiting 148	NCT03161223
Azacitidine Nivolumab INCB059872	DNMT1 PD-1 LSD1	Solid Tumors Hematologic Malignancy (SCLC)	Phase ½	Recruiting 215	NCT02712905
Guadecitabine Atezolizumab	DNMT PD-L1	Chronic Myelomonocytic Leukemia Myelodysplastic Syndrome Recurrent Acute Myeloid Leukemia with Myelodysplasia-Related Changes	Phase ½	Recruiting 72	NCT02935361
Guadecitabine Durvalumab	DNMT PD-L1	Advanced Kidney Cancer Kidney Cancer Clear Cell Renal Cell Carcinoma	Phase ½	Recruiting 48	NCT03308396
Guadecitabine Mocetinostat Pembrolizumab	DNMT HDAC PD-1	Lung Cancer	Phase 1	Recruiting 40	NCT03220477
Anti-PD-1 antibody alone or in combination with decitabine	DNMT PD-1	Multiple Malignancies	Phase ½	Recruiting 250	NCT02961101
Abexinostat Pembrolizumab	HDAC PD-1	Stage III Cutaneous Melanoma, Stage IV Cutaneous Melanoma, Locally Advanced Melanoma Locally Advanced Solid Neoplasm	Phase 1	Recruiting 42	NCT03590054
Entinostat Pembrolizumab	HDAC PD-1	Melanoma	Phase 2	Recruiting 14	NCT03765229
Domatinostat Avelumab	HDAC PD-L1	Gastrointestinal Cancer	Phase 2	Recruiting 75	NCT03812796
Entinostat Pembrolizumab	HDAC PD-1	Myelodysplastic Syndrome	Phase 1	Recruiting 27	NCT02936752
Vorinostat Pembrolizumab	HDAC PD-1	Renal Cell Carcinoma Urinary Bladder Neoplasms	Phase 1	Active, not recruiting 57	NCT02619253
Entinostat Ipilimumab Nivolumab	HDAC CTLA-4	Breast Adenocarcinoma Invasive Breast Carcinoma Metastatic Breast Carcinoma Metastatic Malignant Solid Neoplasm	Phase 1	Active, not recruiting 45	NCT02453620
Romidepsin Pembrolizumab	HDAC PD-1	Colorectal Cancer	Phase 1	Active, not recruiting 27	NCT02512172
Atezolizumab Bevacizumab Entinostat	PD-L1 VEGF HDAC	Advanced Renal Cell Carcinoma	Phase 1/2	Recruiting 62	NCT03024437
CPI-1205 Ipilimumab	EZH2 CTLA-4	Advanced Solid Tumors	Phase 1/2	Not recruiting 24	NCT03525795
Tazemetostat Pembrolizumab	EZH2 PD-1	Locally Advanced Urothelial Carcinoma Metastatic Urothelial Carcinoma	Phase 1/2	Recruiting 30	NCT03854474
Tazemetostat Atezolizumab Obinutuzumab	EZH2 PD-L1	Lymphoma	Phase 1	Completed 96	NCT02220842
BMS: 986158 Nivolumab	BET PD-1	Advanced tumors	Phase 1/2	Recruiting 417	NCT02419417
RO6870810 Daratumumab	BET CD38	Advanced multiple myeloma	Phase 1	Completed 86	NCT03068351

BET = bromodomain and extra-terminal domain proteins, EZH2 = enhancer of zeste homolog 2, IDO1 = indoleamine 2,3-dioxygenase 1, LSD1 = lysine-specific demethylase 1, VEGF = vascular endothelial growth factor.
